# Molecular mechanisms that underpin EML4-ALK driven cancers and their response to targeted drugs

**DOI:** 10.1007/s00018-015-2117-6

**Published:** 2016-01-11

**Authors:** Richard Bayliss, Jene Choi, Dean A. Fennell, Andrew M. Fry, Mark W. Richards

**Affiliations:** 1 Department of Molecular and Cell Biology, University of Leicester, Lancaster Road, Leicester, LE2 9HN UK; 2 Astbury Centre for Structural Molecular Biology, Faculty of Biological Sciences, University of Leeds, Leeds, LS2 9JT UK; 3 Department of Pathology, Asan Medical Center, University of Ulsan College of Medicine, 88 Olympic-ro 43 gil, Seoul, Korea; 4 Cancer Research UK Centre, University of Leicester, Lancaster Road, Leicester, LE3 9SQ UK

**Keywords:** Fusion, Oncogene, Lung cancer, Kinase, Structural biology

## Abstract

A fusion between the EML4 (echinoderm microtubule-associated protein-like) and ALK (anaplastic lymphoma kinase) genes was identified in non-small cell lung cancer (NSCLC) in 2007 and there has been rapid progress in applying this knowledge to the benefit of patients. However, we have a poor understanding of EML4 and ALK biology and there are many challenges to devising the optimal strategy for treating EML4-ALK NSCLC patients. In this review, we describe the biology of EML4 and ALK, explain the main features of EML4-ALK fusion proteins and outline the therapies that target EML4-ALK. In particular, we highlight the recent advances in our understanding of the structures of EML proteins, describe the molecular mechanisms of resistance to ALK inhibitors and assess current thinking about combinations of ALK drugs with inhibitors that target other kinases or Hsp90.

## Background

Lung cancer accounts for around 13 % of all cancers diagnosed worldwide, and accounts for around 1.3 million deaths per year [1]. The most common morphological subtype is lung adenocarcinoma, which is now known to comprise a genomically diverse mosaic of molecular defined subtypes defined by the presence of somatic mutations in so-called driver oncogenes [2]. The prototypical somatic activating mutation in EGFR launched the paradigm shift in personalised medicine for lung adenocarcinoma, [3–5].

Most NSCLC patients are treated with cytotoxic chemotherapy using a DNA-binding drug such as carboplatin/cisplatin in combination with a topoisomerase type II inhibitor such as etoposide or an anti-mitotic such as vinorelbine. Standard treatment also includes immunotherapies that target angiogenesis. Genetic analysis of lung cancers has revealed their underlying complexity, with many different driver mutations that are mutually exclusive in most cases. Molecular subtyping is now routine in NSCLC and patients with an EGFR mutation are treated with an appropriate tyrosine kinase inhibitor (TKI) such as erlotinib in first line therapy. Another reasonably common mutation identified in two landmark studies in 2007 was the first somatic oncogenic gene translocation in lung cancer, involving fusion of two genes, EML4 (echinoderm microtubule associated protein-like 4) and ALK (anaplastic lymphoma kinase) [6, 7]. The identification of EML4-ALK in 4–6 % of lung adenocarcinomas led to the second major clinical development in personalised therapy in NSCLC [8, 9]. Current drug development on EGFR and EML4-ALK have advanced beyond initial therapy, to rational effective treatment upon development of resistance [10, 11]. Efficacy has been demonstrated for kinase inhibitors targeting other rare mutations such as the ROS translocation and BRAF V600E mutation [12, 13].

## Molecular and cellular biology of EML4-ALK fusions

### EML4

**Fig. 1:**
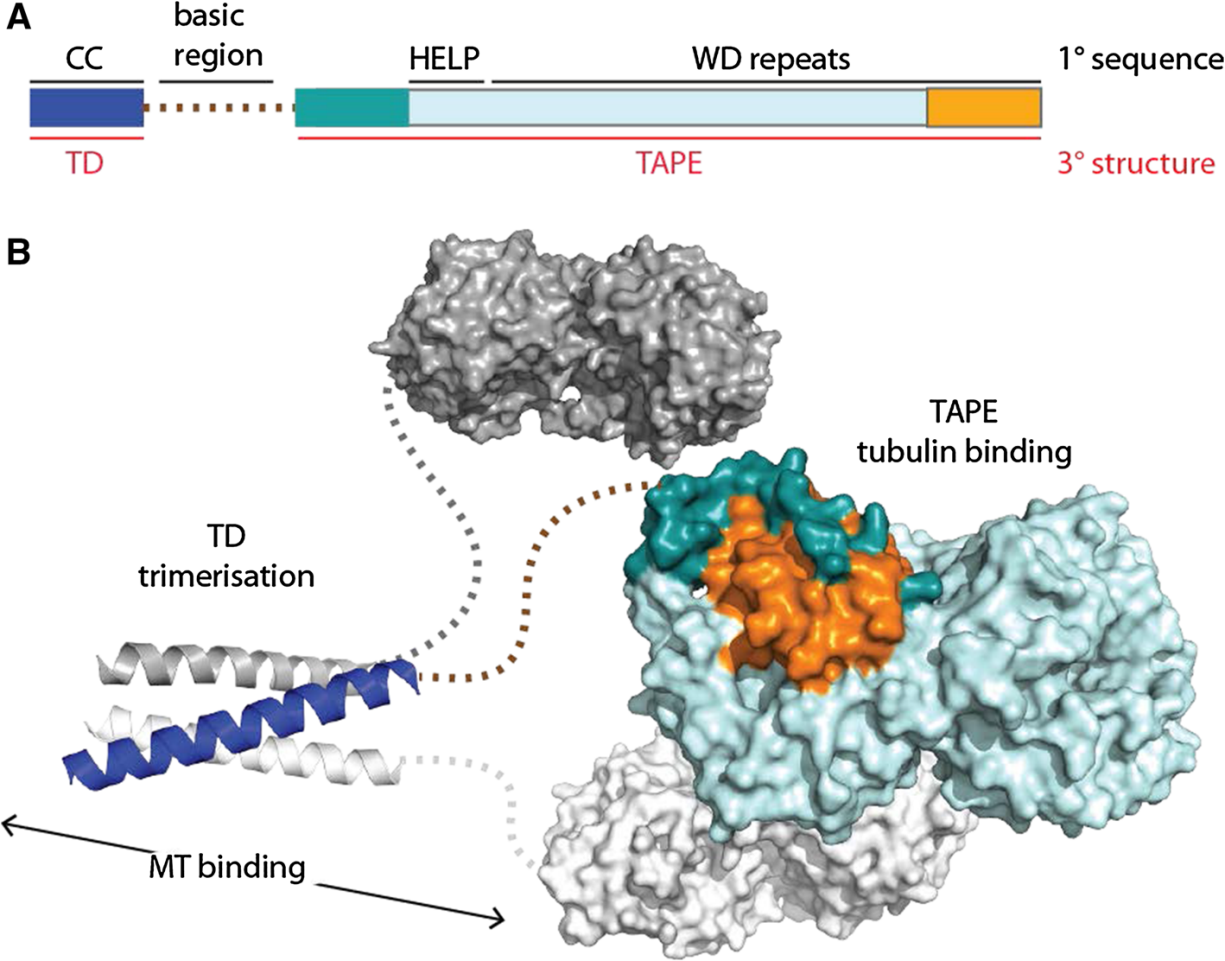
Overview of EML proteins. **a** Domain structure of human EML4 with structural features labelled. Primary (1°) sequence features are labelled in *black*: CC, coiled-coil; basic region; HELP, Hydrophobic motif found in EML proteins; WD repeats, Trp-Asp repeats. Tertiary (3°) structure features are labelled in *red* *text*: TD, trimerisation domain; TAPE, tandem atypical propeller domain found in EML proteins. The TAPE domain N-terminal region is *coloured* *teal* and the C-terminal region is *coloured orange*. **b** Speculative model of the overall trimeric architecture of the EML4 protein. Crystal structures of the EML4 TD (PDB code 4CGC [17]) and EML1 TAPE domain (PDB code 4CI8 [18]) are connected with *dotted lines* to show the basic region that is predicted to be unstructured. One protomer of the trimer is *coloured* using the scheme in 1a—this shows how the N- and C-terminal regions of the TAPE domain (*teal* and *orange*, respectively) come together to close the fold of this domain

Sea urchin EMAP (echinoderm microtubule-associated protein) was the first member of the EML family of proteins to be identified [14]. Subsequently, further EML proteins were found in vertebrates, worms and insects [15]. At the level of primary sequence, the defining feature of EML proteins is the presence of a HELP (hydrophobic EMAP-Like protein) motif N-terminal to a number of WD (Trp-Asp) repeats (Fig. 1a) [16]. The six human EML proteins fall into two classes, based on domain organisation: EML1-4 have an N-terminal coiled-coil region separated from the C-terminal HELP-WD region by a linker region that is rich in Ser, Thr and basic residues, whereas EML5 and EML6 consist of three copies of the HELP-WD region with no coiled-coil.

Recent structural studies have established the molecular architecture of this family of proteins (Fig. 1b). Crystal structures of the coiled-coil regions of human EML2 and EML4 revealed trimeric self-association, and this region was termed the trimerisation domain (TD) [17]. Interestingly, several splice variants of EML2 lack the coiled-coil, resulting in a presumably monomeric protein isoform. The crystal structure of the EML1 HELP-WD region revealed a pair of intimately associated β-propellers [18]. Individual WD40 repeats form a 4-stranded β-sheet that fit together as the individual blades of a propeller structure. Most structures of WD repeat-containing β-propellers are formed from seven blades, but other types occur and the primary sequences of some individual blades do not fit the WD consensus [19]. Due to the uncertainty in predicting WD repeats, the arrangement of two propellers in tandem found in EML1 was unexpected and, atypically, the C-terminal propeller incorporates a blade unrelated to WD40 repeats in both sequence and structure. Moreover, this atypical blade is formed from two sub-domains that originate from distant regions in the primary sequence (coloured orange and dark teal in Fig. 1b). These features of the structure led us to name this novel structure the TAPE (Tandem Atypical Propeller EML) domain.

EML proteins interact with soluble tubulin and microtubules, and a functional association with the mitotic spindle has been shown for several family members [16, 18, 20–23]. Although the HELP motif has been described as a microtubule-binding domain, the crystal structure of EML1 shows that this is not possible because the motif is buried in the interface of the two propellers [18]. In fact, the basic region and TD are necessary and sufficient for microtubule association in the context of EML proteins [17]. Conversely, an intact TAPE domain has the capacity to associate with soluble tubulin. EML4 is expressed in a wide range of tissues, including the lung, and may therefore have a housekeeping role in the formation or maintenance of microtubules [24].

### ALK

**Fig. 2:**
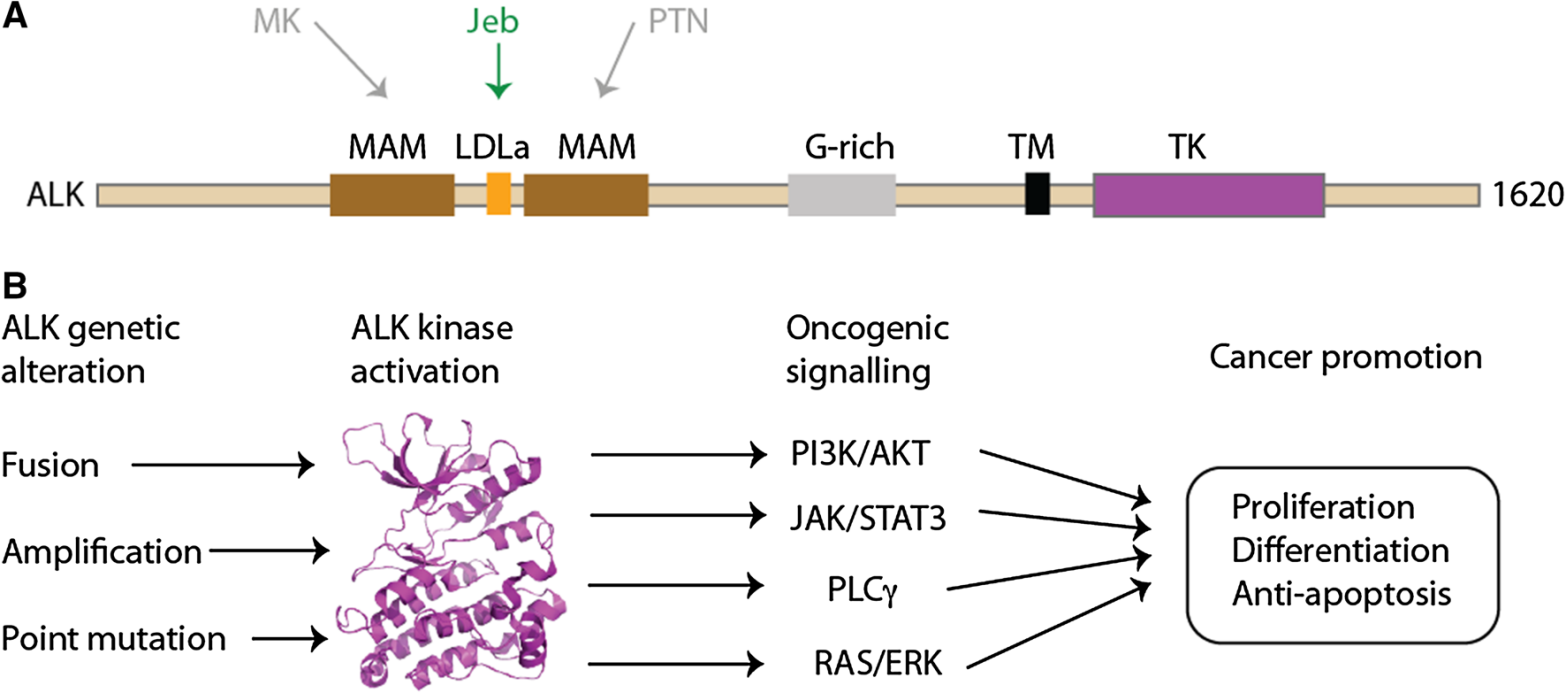
Overview of ALK. **a** Domain structure of ALK with structural features labelled: MAM, Meprin, A5 protein and protein tyrosine phosphatase Mu domain; LDLa, Low-density lipoprotein receptor domain class A; G-rich, glycrine-rich region; TM, transmembrane helix; TK, tyrosine kinase domain. The interactions of upstream, extracellular signalling molecules with ALK are shown: pleiotrophin (PTN) and midkine (MK) as putative ligands in humans; jelly belly (Jeb) as a confirmed ligand in *Drosophila*. **b** Outline of oncogenic ALK signalling mechanisms. Crystal structure of ALK TK domain (PDB code 3LCT [32]) is shown as a *cartoon* representation, *coloured magenta*

ALK is a member of the insulin receptor kinase superfamily, a large grouping which includes other tyrosine kinases of relevance to cancer such as MET and RON [25–28]. ALK was first identified as a fusion partner with nucleophosmin (NPM) in a chromosomal translocation found in non-Hodgkin’s lymphoma, resulting in constitutive kinase activity [29, 30]. ALK is a 1620aa protein with a domain composition that comprises only a few conserved domains interspersed with low complexity sequence that is predicted to be disordered (Fig. 2a). In common with other receptor tyrosine kinases, ALK has a transmembrane (TM) helix and a cytoplasmic TK domain. The extracellular region includes two MAM (Meprin, A5 protein and protein tyrosine phosphatase Mu) domains, one on either side of a LDLa (Low-density lipoprotein receptor domain class A) domain, all of which are N-terminal to a Gly-rich region that precedes the TM helix. The structure of the TK domain has been resolved using X-ray crystallography, but there are currently no structures of the other domains of ALK [31, 32].

Based on its expression pattern, ALK most likely contributes to the development of the nervous system [33, 34]. Knockout mice are viable and show no gross defects but have behavioural abnormalities [35, 36]. Activating ligands of ALK in model organisms such as *D. melanogaster* and *C. elegans* have been identified and the contribution of ALK to the development of specific tissues has been elucidated [37]. For example, in *Drosophila*, the secreted protein ligand jelly belly (Jeb) activates Alk kinase in synaptogenesis and the organisation of muscle tissue [38–40]. The identification of activating ALK ligands in humans is inconclusive and although pleiotrophin (PTN) and midkine (MK) have been proposed [41–43]; there is no consensus on whether these are bone fide activators of ALK [37, 44].

Inarguably, the pathways through which mutant ALK mediates oncogenesis are more comprehensively explored. ALK signalling is activated in cancer cells through three principal mechanisms: gene fusion events, such as NPM-ALK and EML4-ALK; ALK gene amplification; and activating point mutations such as F1174L in neuroblastoma (Fig. 2b). The wider picture of ALK activation has been expertly reviewed elsewhere [37]. In ALK fusions, the partner drives ALK activity at the level of gene expression and through multimerisation of the ALK kinase domain, which is presumed to promote autophosphorylation. Furthermore, the partner determines the sub-cellular localisation of the fusion protein. The oncogenic potential of ALK fusions has been demonstrated in cell and animal model systems [7, 45–47].

The pathways through which ALK mediates oncogenesis have been extensively explored in the case of NPM-ALK (reviewed by [37, 48]). NPM-ALK activation signals through multiple pathways, including RAS/ERK, PLCγ [46], PI3 K/AKT, and JAK/STAT [49]. These confer neoplastic phenotype, cell proliferation, cell survival and changes in cytoskeleton. In contrast to the extensive studies on NPM-ALK, there has been relatively little experimental validation of signalling pathways mediated by EML4-ALK, which has been done mainly through the use of ALK inhibitors in cell lines derived from NSCLC patients and model cell lines expressing recombinant EML4-ALK.

### Molecular genetics of EML4-ALK patients

**Fig. 3:**
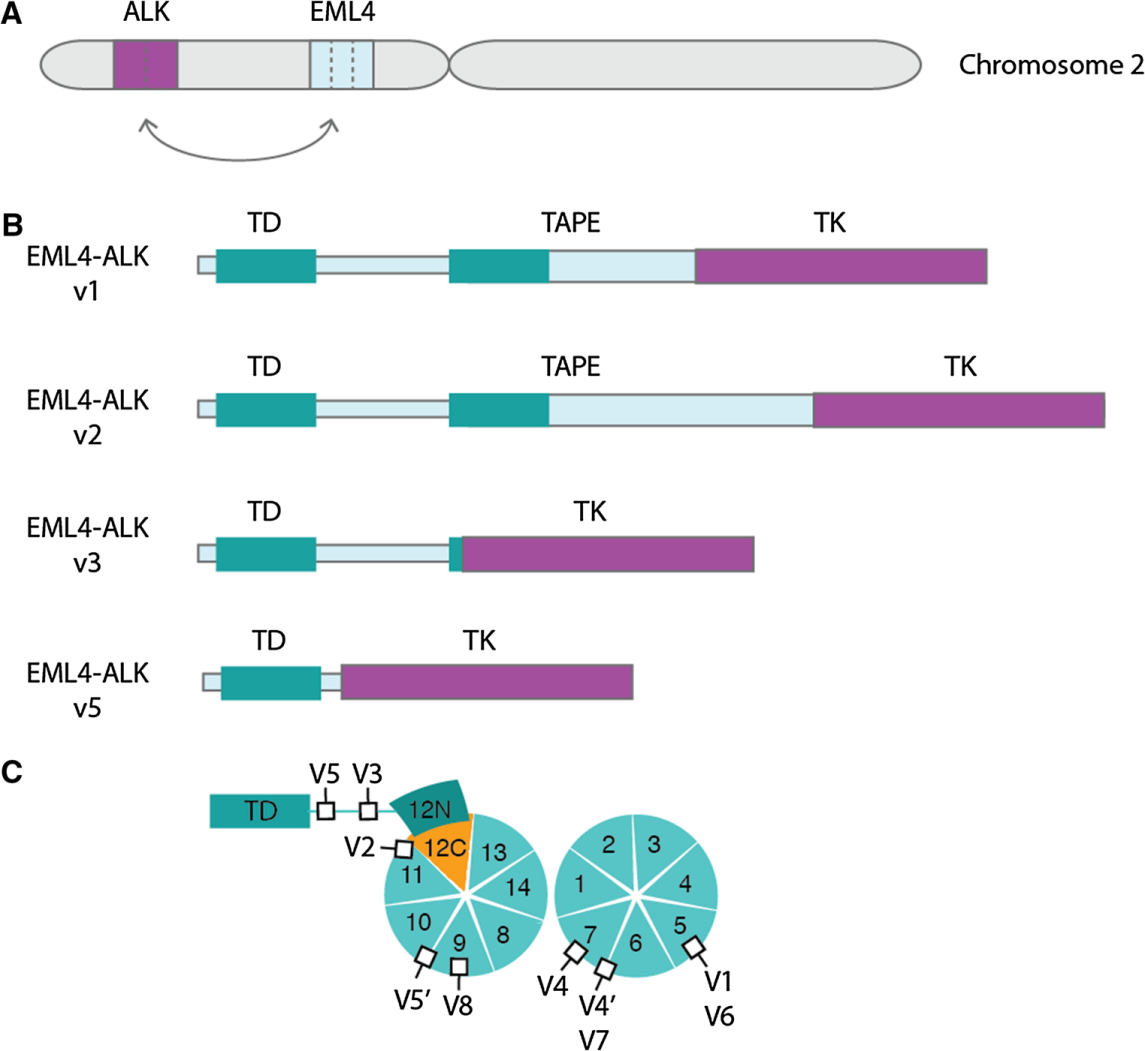
Overview of EML4-ALK fusions and variants. **a** EML4-ALK fusion gene is generated by a paracentric inversion on the short arm of chromosome 2. *Dotted lines* indicate potential fusion sites. **b** Schematic illustrations of four major EML4-ALK variant proteins, showing where the ALK TK domain is inserted into the EML4 protein. **c** The individual subdomains that make up the TAPE domain of EML4 are shown. The two propellers of the TAPE domain have thirteen canonical blades and a non-canonical blade comprising the 12N and 12C subdomains. The positions of ALK TK domain insertion into the EML4 structure are shown as *black squares*, labelled with the variant number. Note that, with the exception of v3 and v5, all variants have ALK inserted into the TAPE domain

The EML4 and ALK genes are oriented in opposite directions within the short arm of chromosome 2, and the EML4-ALK fusion gene arises through a paracentric inversion of this region [inv(2)(p21p23)] (Fig. 3a) [7]. In all cases, the breakpoint within the ALK gene lies close to the 5′ end of exon 20. Thus, the entire extracellular domain and TM helix are excluded from the EML4-ALK fusion, which just incorporates the cytoplasmic portion of ALK including the TK domain. The fusion points within the EML4 gene are more variable. The most common variants end at exon 13 (v1), exon 20 (v2) and exon 6 (v3) [7, 50, 51]. At least fifteen variants have been identified, the shortest of which, v5, includes just exons 1 and 2 of EML4 (Table 1).

**Table 1:** List of EML4-ALK variants

EML4-ALK variant	Gene fusion points	Mutation frequency^1^	Cell lines	Structural features	References
V1	E13;A20	33 % (25 %)	H3122, DFCI032, STE-1	EML4- TD, basic, HELP motif, incomplete TAPE ALK TK	[7, 6, 51, 55]
V2	E20;A20	9 % (7.5 %)		EML4- TD, basic, HELP motif, incomplete TAPE ALK TK	[7, 51]
	E20;ins18A20	(<1 %)		EML4- TD, basic, HELP motif, incomplete TAPE ALK TK	[108]
V3a	E6a;A20	29 % (18.5 %)^2^	H2228	EML4- TD, basic No HELP or TAPE ALK TK	[51]
V3b	E6b;A20	29 % (18.5 %)^2^	H2228	EML4- TD, basic No HELP or TAPE ALK TK	[6, 51]
V4	E15del60;del71A20	2 % (<1 %)		EML4- TD, basic, HELP motif, incomplete TAPE ALK TK	[51]
V4′	E14;ins11del49A20	3 % (<1 %)^3,5^		EML4- TD, basic, HELP motif, incomplete TAPE ALK TK	[52]
V5a	E2;A20	2 % (<1 %)^4^		EML4- TD No basic, HELP or TAPE ALK TK	[52]
V5b	E2;ins117A20	(<1 %)		EML4- TD No basic, HELP or TAPE ALK TK	[52]
V5′	E18;A20	2 % (<1 %)		EML4- TD, basic, HELP motif, incomplete TAPE ALK TK	[109]
V6	E13;ins69A20	(<1 %)		EML4- TD, basic, HELP motif, incomplete TAPE ALK TK	[110]
V7	E14;del12A20	See V4′ (<1 %)^3,5^		EML4- TD, basic, HELP motif, incomplete TAPE ALK TK	[110]
V8a	E17;ins30A20	(<1 %)^6^		EML4- TD, basic, HELP motif, incomplete TAPE No functional ALK domain	[111]
V8b	E17ins61;ins34A20	(<1 %)^6^		EML4- TD, basic, HELP motif, incomplete TAPE ALK TK	[111]
	E17;ins68A20	1 % (<1 %)^6^		EML4- TD, basic, HELP motif, incomplete TAPE ALK TK	[108]
Unknown		19 % (45 %)			

^1^Mutation frequency obtained from [53] and COSMIC (Catalogue of somatic mutations in cancer), shown in parentheses [112]

^2^Note that frequency of V3a and V3b are the same because they are splice variants

^3^Note that V4′ and V7 are combined in the source Ref. [53]

^4^Note that V5a and V5b are combined in the source Ref. [53]

^5^Note that COSMIC lists E14;A20 translocations, but not V4′ and V7

^6^Note that COSMIC lists E17;A20 translocations, but not V8a or V8b

Both v3 and v5 exist as a mixture of two isoforms generated by alternative splicing [51–53]. These variants of EML4-ALK fusion proteins all have a TD, which is essential for ALK autophosphorylation and activation. Most variants have the basic region and many have a variable portion of the TAPE domain (Fig. 3b). The presence of incomplete protein domains in some EML4-ALK variant proteins is unusual because oncogenic gene fusion proteins normally incorporate only intact domains connected by linker regions [54]. This feature has consequences for the biology of EML4-ALK fusion proteins, their molecular properties, the pathways through which they signal and potential therapeutic approaches.

### EML4-ALK cell lines and signalling

Cell lines harbouring EML4-ALK fusions were established from NSCLC patients [51, 55]. Variant 1 was detected in H3122, DFCI024 and STE-1 cell lines, whereas variant 3a/b was found in the H2228 cell line. These cell lines have formed the basis for most of the studies on the signalling pathways downstream of EML4-ALK, EML4-ALK drives the phosphorylation of Akt and ERK, which are reduced upon ALK inhibition (TAE684) in H2228 and H3122 cell lines [51, 56, 57]. EML4-ALK is upstream of ERK in Ba/F3 cell lines expressing EML4-ALK fusions, but does not seem to influence Akt in this system [58]. Ba/F3 is a murine pro-B cell line that depends on IL‐3 for cell growth and this dependency is overcome when certain oncogenes are expressed, such as EGFR and EML4-ALK [7, 59, 60]. EML4-ALK may also be upstream of STAT3 phosphorylation; however, there is no clear consensus among reports. While some studies show a clear reduction in STAT3 phosphorylation upon TAE684 treatment [57, 61], other studies show a minimal effect of TAE684 on STAT3 phosphorylation in H2228 and H3122 [51].

There are differences in sensitivity towards ALK inhibitors between EML4-ALK variants. For example, the growth of H3122 cells is sensitive to TAE-684, whereas H2228 and DFCI032 are only a little more sensitive than controls (A549—KRAS G12S, PC-9—EGFR del E746-750) [51]; however, other groups found that both H3122 and H2228 cells are sensitive to TAE-684 [57] or that while TAE684 has a cytotoxic effect on H3122, there is a cytostatic effect on H2228 [56]. Upon addition of TAE-684, EML4-ALK phosphorylation is ablated in all three cell lines harbouring the fusions, however, the consequences for downstream signalling pathways are divergent: p-Akt and p-ERK are reduced in H2228 and H3122, but these phosphorylation signals are lost entirely only in the H3122, which exhibit PARP cleavage and apoptosis [56, 61]. In contrast, inhibition of ALK alone is not sufficient to induce significant apoptosis in H2228 cells. Using the Ba/F3 model, sensitivity of variants to two chemically-distinct inhibitors (TAE684 and crizotinib) showed a similar pattern—v2 most sensitive, v1 and v3b intermediate and v3a least sensitive, covering a GI_50_ range from 0.15 to 1 μM [58]. This was not due to differences in potency for the variants, but differential downstream responses, notably in ERK phosphorylation. Finally, ALK inhibition destabilised the fusion proteins but to differing degrees in that v2 and, to a lesser extent, v1 are destabilised by ALK inhibitor, whereas this effect is less clear in v3a [58].

ALK activation is driven by the TD of EML4, which drives self-association of the kinase domains. The TAPE domain is not critical for ALK activation because deletion within the TD and basic regions (aa31-140) of EML4-ALK v1 reduced kinase activity and transforming activity, but deletions within the TAPE domain had little or no effect [7]. Indeed, EML4-ALK v5, which incorporates the shortest portion of EML4 of any fusion and lacks any of the TAPE domain, also has kinase and transforming activity. Whilst the TD domain could drive autophosphorylation following self-association of the kinase domain, the mechanism by which ALK is activated by the TD is unknown and it is unclear how it might differ from ALK fusions in which the partner is dimeric. In addition, from a structural biology point of view, it seems likely that the mechanistic details of ALK activation might be influenced by the presence of portions of the TAPE domain.

The intracellular localisation of the most common EML4-ALK variant proteins has been studied in model cell lines and in cancer cells derived from patients. Using recombinant proteins expressed in NIH3T3 cells, EML4-ALK v1 and v2 were localised to the cytoplasm, whereas v3 localised to the nucleus, using an ALK antibody to label the fusion proteins [58]. YFP-fusions of EML4-ALK v1, v2 and v5a expressed in HeLa had a diffuse, cytoplasmic localisation, whereas v3a co-localised with microtubules in the cytoplasm [17]. Consistent with the localisation of the recombinant proteins, endogenous EML4-ALK v1 was diffusely localised in the cytoplasm in H3122 cells and endogenous EML4-ALK v3a co-localised with microtubules in H2228 cells [17]. Independent studies of the localisation of endogenous EML4-ALK v1 to the cytoplasm of H3122 and STE-1 cells showed a more discrete pattern of ALK puncta, described as an unidentified intracellular compartment [62]. It appears that the majority of EML4-ALK fusion protein variants do not associate strongly with microtubules, despite having the basic region and TD of EML4. Further studies are required to investigate the localisation of EML4-ALK fusions at higher resolution and to clarify whether microtubule association is relevant to ALK signalling, as is the case for other RTK fusions with microtubule binding proteins [63].

## EML4-ALK patients and their treatment

NSCLC patients harbouring EML4-ALK or EGFR mutations are younger, have more advanced disease and are more likely to be non-smokers or light smokers than the average [53]. Furthermore, EML4-ALK fusions are more common in lung adenocarcinoma than other NSCLCs [64]. NSCLC patients with EGFR mutations show a markedly improved response when treated with TKIs such as gefitinib, with a doubling of the time to progression (TTP) and greater than 50 % increase in overall survival [4, 65]. EML4-ALK NSCLC patients do not generally have EGFR mutations or indeed any other key driver mutations, such as RAS [7, 65, 66]. There are of course exceptions: recent reports described an EML4-ALK patient lacking EGFR mutation who responded to erlotinib [67], and a patient who harboured both EML4-ALK and EGFR mutations [68] potentially reflecting intratumour genomic heterogeneity which may in some cancers be considerable [69]. There have been intensive efforts to develop ALK TKIs, and EML4-ALK patients receiving them exhibit superior response rates and disease control compared with chemotherapy [9].

### ALK inhibitors

**Fig. 4:**
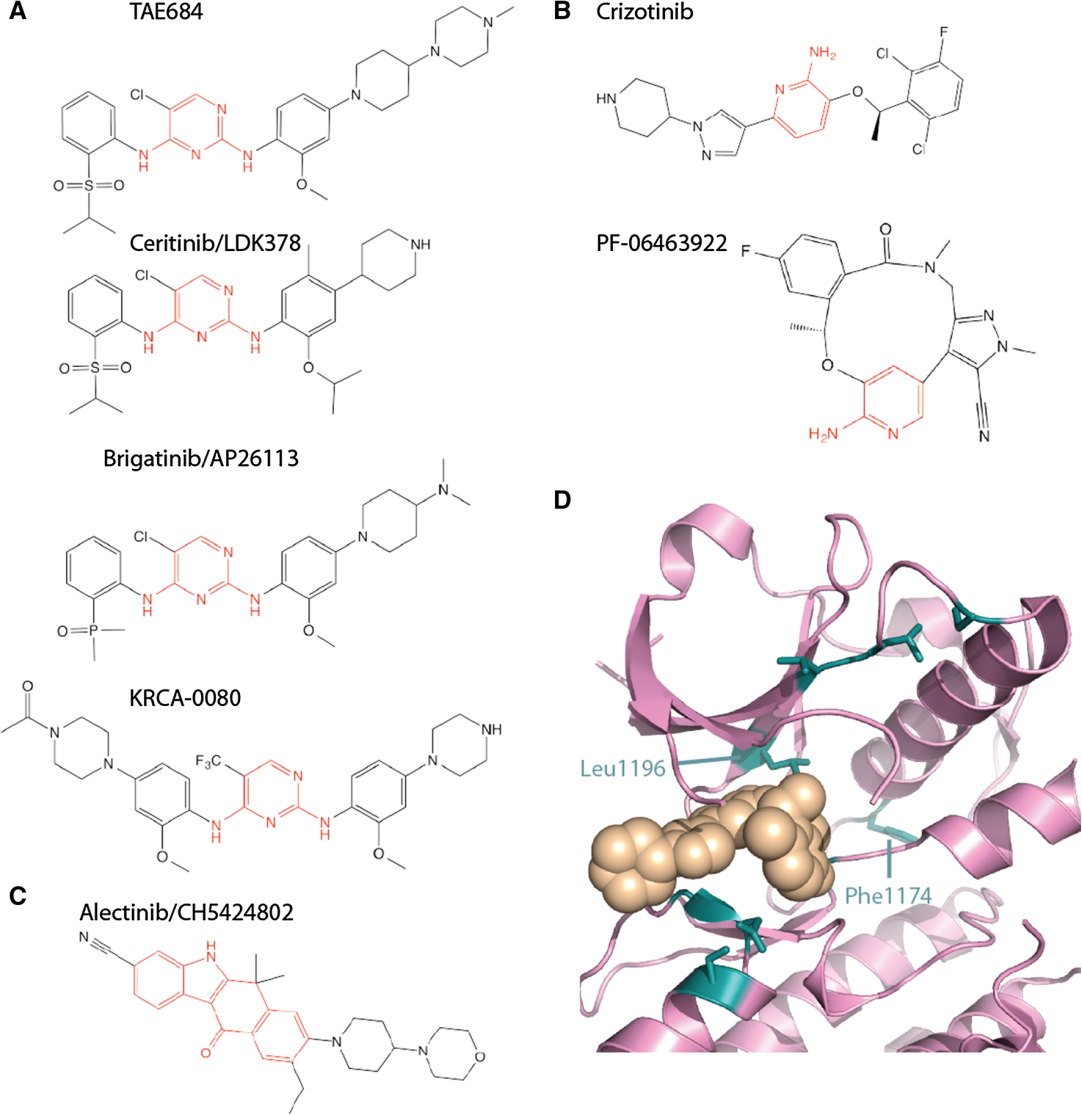
ALK tyrosine kinase inhibitors. Chemical structures of ALK TKIs are shown with the core scaffold highlighted in *red*. **a** diaminopyrimidine. **b** Aminopyridine. **c** Dihydrobenzocarbazole. **d** Crystal structure of ALK TK domain (*pink* *cartoon*) bound to crizotinib (*beige spheres*). The image is based on PDB code 2XP2 [71]. Key residues mutated in drug resistance ALK are shown as *sticks*, *coloured* *teal*

The first research papers on EML4-ALK used non-specific inhibitors such as WHI-P154 [7] or TAE684 [51] (Fig. 4a). However, the first clinical studies used crizotinib, a selective ALK/MET/ROS ATP-competitive TKI that is orally available and was evaluated in patients with advanced solid tumours (Fig. 4b) [65]. EML4-ALK NSCLC patients showed clear responses, and an expanded trial (PROFILE 1001) was established that focussed on EML4-ALK patients. The results of this trial were positive, with over 60 % overall response rate, leading to a phase II trial (PROFILE 1005) with a similar rate of response. On the basis of these trials, crizotinib was approved by the FDA in 2011 for the first-line treatment of advanced NSCLC in patients with ALK+ tumours. In other countries, there have been concerns over the cost of treatment relative to the benefit and in the UK crizotinib is available to patients in the UK through the cancer drugs fund. A further clinical study compared the efficacy of crizotinib to additional chemotherapy (docetaxel or pemetrexed) in patients who had already undergone one round of platinum-based chemotherapy (PROFILE 1007). Crizotinib was better in terms of progression-free survival and overall response rate, but not in overall survival. Initial results from an ongoing phase III trial in a first-in-line setting shows that crizotinib is superior to chemotherapy (PROFILE 1014) in terms of progression-free survival and overall response rate, but it is too early to know whether there is a significant difference in terms of overall survival [70].

Many of the available ATP competitive ALK inhibitors, including the clinically approved drugs and those under investigation, are based on three chemical scaffolds (Fig. 4a–c)—aminopyridine (crizotinib and PF-06463922) [71, 72], diaminopyrimidine (TAE684, ceritinib, brigatinib, KRCA-0080) [73, 74] and the unusual dihydrobenzocarbazole (alectinib) [75]. PF-06463922, an ATP-competitive ALK/ROS1 inhibitor is a macrocyclic compound, based on stabilization of the closed conformation of crizotinib bound to ALK that was observed in the crystal structure (Fig. 4b, d) [72].

### Mechanisms of resistance to ALK TKIs

EML4-ALK patients who initially respond to TKIs such as crizotinib inevitably relapse as the tumour acquires resistance to the drug. Many different resistance mechanisms have been identified, the most common of which is alterations in the EML4-ALK gene, which occurs in approximately a third of patients. These alterations are primarily EML4-ALK gene amplification, mutations within the TK domain of ALK (Table 2), or sometimes both [76–80]. Another frequently documented drug resistance mechanism is upregulation of alternative RTKs, such as EGFR, KIT, IGF-1R and SRC, to activate bypass pathways [55, 77–79, 81]. However, these mechanisms do not account for all drug resistance. To address this shortfall in our knowledge, a recent study screened 12,800 human ORFs to identify genes that confer resistance to crizotinib or TAE684 in H3122 cells [82]. The screen identified a diverse selection of 54 genes, including kinases, growth factors, scaffolding proteins and DNA-associated proteins, half of which were able to drive ERK and/or AKT activity. Among the genes were P2 purinergic receptors of the P2Y subfamily, which activate protein kinase C (PKC) to mediate resistance to crizotinib [82]. The major effort to identify and characterise drug resistance pathways that bypass ALK and to develop strategies for overcoming resistance in patients will continue. However, at present, there is more substantial progress in addressing the challenge of ALK-dependent drug resistance mechanisms.

**Table 2:** Drug sensitivity or resistance to ALK mutations

ALK inhibitor	Crizotinib	Ceretinib	Alectinib	PF-06463922
*Resistance mutation*				
G1123S		Resistant [113]	Sensitive [113]	
1151Tins	Resistant [79, 90]	Resistant [90]	Resistant [79]	Sensitive [87]
L1152P/R	Resistant [90]	Resistant [90]	Sensitive [114]	Sensitive [87]
C1156Y/T	Resistant [90]	Resistant [90]	Sensitive [87]	Sensitive [87]
I1171T/N	Resistant [90]	Sensitive [90, 115, 116]	Resistant [115, 116]	Sensitive [87]
F1174L/C	Resistant [90]	Resistant [90]	Sensitive [115]	Sensitive [87]
V1180L	Resistant [115]	Sensitive [115]	Resistant [115]	Sensitive [87]
L1196M	Resistant [78, 79, 83]	Sensitive [90]	Sensitive [87]	Sensitive [87]
G1202R	Resistant [79, 90]	Resistant [84, 90]	Resistant [79]	Sensitive [72, 87]
S1206Y	Resistant [79, 90]	Sensitive [90]	Sensitive [79]	Sensitive [87]
G1269A/S	Resistant [90]	Sensitive [90]	Sensitive [114]	Sensitive [87]

Drug-resistant mutations in the ALK TK domain cluster around the ATP binding site and enhance ATP binding, reduce crizotinib binding or both (Fig. 4d; Table 2) [80]. The most common mutation is residue Leu1196 to methionine [78, 83, 84]. Leu1196, sometimes referred to as the gatekeeper, lies at the back of the active site and forms extensive interactions with crizotinib. The mutation disrupts these interactions, weakening the potency of crizotinib [76]. The gatekeeper position in the active site is frequently the site of drug-resistant mutations in other tyrosine kinases, such as T315I in ABL and T790M in EGFR that disrupt drug interactions and help to activate the kinase [85]. Acquired drug-resistant mutations are also observed in Phe1174, which is the site of driver mutations in ALK in neuroblastoma [86]. One route to overcoming drug resistance is to use an alternative drug that is more potent against mutant ALK. Indeed, a subset of the mutations that desensitize ALK to crizotinib retain sensitivity to the second-generation ALK inhibitors alectinib and ceretinib (Table 2). However, the 1151Tins and G1202R mutations confer resistance to all second-generation inhibitors.

The macrocylic inhibitor PF-06463922 is more potent against WT ALK and ALK mutants than crizotinib and retains substantial activity against ALK mutants that are resistant to crizotinib and second-generation inhibitors [87]. Although activity against G1202R ALK is lower than WT (77 nM IC50 vs 1.3 nM IC50), this is similar to the potency of crizotinib against WT ALK [72]. Furthermore, PF-06463922 is active against alectinib-resistant cell lines harbouring the I1171T or V1180L mutant, and ceretinib-resistant cell lines harbouring G1202R [87]. PF-06463922 elicits a durable response in mouse xenograft models of EML4-ALK NSCLC, based on H3122 cell lines harbouring WT ALK or crizotinib-resistant mutants L1196 M and G1269A, and the compound induces tumour regression in mice bearing tumours that relapsed on crizotinib. Brain metastasis is the first step in disease progression in up to 50 % of NSCLC EML4-ALK patients being treated with crizotinib [88]. Importantly, PF-06463922 is predicted to cross the blood–brain barrier in humans, and is active against an NSCLC brain metastasis model using intracranial xenografts of WT or L1196M ALK H3122 cells [87]. This is an improvement over crizotinib, which has limited CNS penetration, and the brain is the most common site of relapse of patients treated with crizotinib [89]. Ceretinib also has brain penetration and is approved in patients who have developed resistance to crizotinib [90].

The development of ATP-competitive inhibitors has improved outcomes for patients and there is scope for further improvements using the newest ALK inhibitors. It is, however, inevitable that drug resistance will emerge for these compounds and alternative strategies are under investigation that could be applied as second-line to or in combination with ALK inhibitors.

### Hsp90 inhibitors

Molecular chaperones are a structurally diverse group of proteins that function in protein folding and quality control [91, 92]. Hsp90 is an ATP-dependent chaperone that stabilises a wide variety of client proteins, including many proteins that function in cancer-relevant pathways [93]. Geldanamycin and radicicol, both natural product inhibitors of Hsp90, suppress oncogenic transformation but are too unstable to be used clinically [94, 95]. More stable derivatives of geldanamycin, such as 17-AAG, 17-DMAG and IPI-504 and synthetic Hsp90 inhibitors, such as NVP-AUY922 and ganetespib, have been developed and tested clinically in a number of cancers [93, 96, 97]. These inhibitors act by blocking the ATP binding site of Hsp90 and halting the ATPase cycle [91, 98, 99].

The first evidence that Hsp90 inhibitors might be useful in EML4-ALK patients came from a phase II study that assessed the efficacy of IPI-504 after EGFR TKI therapy in patients with advanced NSCLC [100]. Although there was a low response rate among patients with EGFR mutations, strikingly, all three patients harbouring EML4-ALK fusions responded, two with partial response and one with stable disease over 7.2 months. EML4-ALK NSCLC cell lines H3122 and MGH006 (variant unpublished) were more sensitive to Hsp90 inhibition than EGFR mutant cell lines, and the fusion protein was destabilized upon Hsp90 inhibitor treatment. This work was followed up in further studies that showed EML4-ALK to be among the most sensitive Hsp90 client proteins and crizotinib-resistant H3122 (CR) (crizotinib-resistant) cells retained sensitivity to Hsp90 inhibition [101, 102]. More recently, ganetespib was shown to be effective at reducing tumour growth in mice bearing H3122 cell xenografts, by ~80 to 90 % after 33 days post-implantation [103]. This raises the question of why EML4-ALK is so sensitive to Hsp90 inhibition, and how this property might be exploited in treatment.

Not all EML4-ALK fusion variants are equally sensitive to Hsp90 inhibition as v1 and v2 but not v3a were found to be sensitive to 17-DMAG [58]. Furthermore, artificial EML4-ALK fusions based on v2 lacking additional one additional WD repeat was equally sensitive to 17-DMAG, but removing 5 WD repeats or the entire HELP-WD region gave rise to fusions that are as stable as control cells [58]. It was concluded that the molecular basis of EML4-ALK sensitivity to Hsp90 inhibitors is a property of disruption of the WD repeat region [58]. Models of EML4-ALK fusion protein variants based on the crystal structure of the equivalent region from EML1 provided a structural explanation for these observations [18]. In variants that are more sensitive to Hsp90 inhibition, such as v1 and v2, the fusion breakpoint disrupts the TAPE domain, resulting in a protein with insufficient number of WD repeats to form complete propeller structures. These proteins are highly unstable and dependent on Hsp90. Variants such as v3 and v5, which lack the TAPE domain entirely, are independent of Hsp90. H3122 cells harbouring v1 are more sensitive to the Hsp90 inhibitor ganetespib than H2228 cells harbouring v3, and a similar observation has been made using 17-DMAG [18, 104]. It is important to establish whether the difference in Hsp90 inhibitor sensitivity observed in vitro is a factor in the response of patients to these inhibitors so that informed decisions can be made regarding the most effective treatment strategy for patients with tumours harbouring the different EML4-ALK fusion variants.

Because they act through a different mechanism, Hsp90 inhibitors also induce the degradation of ALK fusion proteins that are resistant to ALK inhibitors. For example, ganetespib is equally potent against 15 different drug-resistant mutants of NPM-ALK fusions as wild-type NPM-ALK [103]. Furthermore, Hsp90 inhibitors do not just deplete EML4-ALK, and their ability to block multiple oncogenic pathways may be advantageous in patient treatment. Several oncogenic RTKs are Hsp90 clients, and Hsp90 inhibitors could prevent drug resistance arising through bypass pathways mediated by these RTKs. For example, EGFR and MET activation by addition of ligands induces resistance to alectinib in H2228 cells, which can be overcome with Hsp90 inhibition [104]. Resistance to Hsp90 inhibitors could be mediated through upregulation of other heat shock proteins, such as Hsp70, or through alterations in apoptotic signalling pathways, and it remains to be seen which mechanisms contribute in NSCLC patients [105, 106]. Hsp90 inhibitors may therefore be particularly useful in the treatment of NSCLC patients who have developed resistance to ALK inhibitors, or perhaps as a first-line therapy in combination with ALK inhibitors.

### Combinations of ALK and inhibitors of other RTKs or Hsp90

Cytotoxic chemotherapy treatment is usually delivered as a combination of drugs, which maximize cancer cell death through additive effects or synergism between mechanisms of action. By this rationale, it should be possible to find combinations of ALK inhibitors with other therapeutics that improve patient outcomes. There are many ways to identify combinations of drugs, starting from a rational basis or through screening approaches.

Synergy between ALK and Hsp90 inhibitors has been shown in Ba/F3 cells, but was dependent on the EML4-ALK fusion variant with v1 showing the least effect [58]. The combination of crizotinib and ganetespib showed enhanced cell death of H3122 cells in culture, and the combination reduced tumour growth by over 90 % in mice bearing H3122 xenografts [103]. Clinical trials of ALK and Hsp90 inhibitors in combination are underway.

Insulin-like growth factor 1 receptor (IGF-1R) was identified as a potential target for combinatorial drug treatment of EML4-ALK NSCLC, based on the exceptional response of a patient to IGF-1R mAb plus erlotinib and an in-depth study of EML4-ALK cell lines [55]. The patient’s tumour was not molecularly profiled until she progressed on this treatment, when an ALK rearrangement was identified and she was treated with crizotinib. Inhibition of ALK using crizotinib and IGF-1R was synergistic in EML4-ALK model cell lines (H2228, H3122 and STE-1), and in crizotinib-resistant cell lines based on H3122. IGF-1R pathway activity was increased in crizotinib-resistant cell lines. Interestingly, the authors of this study noted that the LDK-378 ALK inhibitor also inhibits IGF-1R, which may in part explain the high response rate to this drug of patients who have progressed on crizotinib [55].

Several signalling pathways are activated in EML4-ALK cells, and dual targeting of ALK with these other pathways may be beneficial, particularly in pathways that are not entirely blocked by ALK inhibition. For example, treatment of H2228 cells with TAE684 does not markedly affect phosphorylation of ERK at concentrations at which ALK phosphorylation is inhibited [56, 61]. Indeed, a combination of ALK and MEK inhibitors substantially increases apoptosis in H2228 cells [61].

A recent paper provides evidence that upfront dual inhibition of ALK and MEK may be advantageous, at least in some EML4-ALK patients [62]. Cell growth of H3122 or STE-1 cells was decreased by inhibition of ALK or MEK, but not inhibition of AKT or PI3 K. Lung cancer cell lines harbouring K-RAS or BRAF mutations, but not EGFR, were also sensitive to MEK inhibition. Activation occurs at the level of RAS because GTP loading of N-RAS, H-RAS and K-RAS were all enhanced by EML4-ALK in H3122 or STE-1 cells. The mechanism of RAS activation by EML4-ALK is unclear because the fusion protein does not localise to the plasma membrane. Intriguingly, RAS activation appears to be dependent on the HELP motif of EML4-ALK v1. This implies that EML4-ALK variants lacking the HELP motif, such as v3 and v5, may have different downstream signalling, perhaps as a consequence of their association with distinct complexes and alternative subcellular localisations.

The combination of ALK and MEK inhibitors was also effective in a ceritinib-resistant cell line derived from a patient and, in this case, the effectiveness of the combination may have been due to the presence of an activating mutation in MAP2K1 [81]. However, in this study, there was no advantage to combining the two inhibitors in other cell lines. In contrast, a different study suggests that a high proportion of the bypass resistance mechanisms in response to ALK inhibition involve activation of MAPK signalling [82]. There are several other candidate kinase targets for co-inhibition alongside ALK, including SRC, ERBB2/HER2 and PKC [51, 81, 82]. It is not yet clear which combinations of kinase inhibitors should be prioritized for clinical studies and how patients should be selected for these trials.

## Conclusions

The identification of the EML4-ALK fusion in a subset of NSCLC patients, and the response of these patients to crizotinib is a clear example of the value of molecular genetics aligned with targeted approaches to cancer drug discovery. The ability of researchers to develop second-generation ALK inhibitors that have substantially improved pharmacological properties is a testament to the power of medicinal chemistry, backed by rational drug design approaches. However, as exciting as these advances are, we are still a long way from being able to offer patients a programme of treatments that cures their disease, or at least keeps it at bay for a decade or more. Therefore, the key challenge ahead is to identify rational combinations of drugs based on molecular and genetic analysis of an individuals tumour that prolong the duration of response to treatment, and to identify appropriate strategies to overcome resistance. Here we have described the current state of the art, from which improved programmes of treatment will develop. We believe that the answers to the following questions will be critical if we are to achieve dramatically improved patient outcomes:

*What are the missing connections in the signalling pathways that connect EML4*-*ALK to cancer proliferation and survival, and how can we exploit these in therapy?* Over the next few years, we will discover much more about this intriguing oncoprotein and how the combination of different portions of EML4 and ALK affect its behaviour. In particular, we will learn much about the signalling pathways and mechanisms of resistance from clinical studies on second-generation ALK inhibitors alone and in combination with other therapeutics.

*What is the best strategy for ALK inhibitors in the clinic?* Second-generation ALK inhibitors show benefit in patients who have relapsed on crizotinib, and have been approved by the FDA for treatment of these patients. We do not yet know which of them are the preferred options in crizotinib-resistant patients and whether, in time, they ought to replace crizotinib as the first-line therapy.

*Do we need to develop further ALK inhibitors, and what properties should they have?* Unfortunately, drug resistance to advanced ALK inihibitors is inevitable. Because these inhibitors are more potent against ALK, and retain effective potency against key ALK mutants, we would expect a higher proportion of mutations that activate bypass pathways versus further mutations in ALK or ALK overexpression. In this context, the off-target effects of crizotonib, such as inhibition of MET, AXL and RON, may be beneficial in preventing the activation of bypass resistance pathways [82]. In considering the development of further ALK inhibitors to fully address drug resistance mechanisms, activity against selected other kinases may be a desirable property, as well as activity against key drug-resistance mutations in ALK.

*How are bypass pathways selected for during ALK inhibitor treatment, can we predict which path of drug resistance a tumour is likely to exploit, and how do we target these pathways on an individual patient basis?* Initial studies suggested a number of bypass pathways, and we have to build a clear picture of the overall signalling network. Addressing this, for example through next generation sequencing, will require significant effort over the next few years as clinical samples of patients treated with second-generation ALK inhibitors become available.

*Which therapeutics should be used in addition to ALK inhibitors and how?* There are many suggestions for therapeutics that could be used following ALK inhibitory therapy, or perhaps in combination. These include Hsp90 inhibitors and inhibitors of other RTKs. It is also possible that cancer immunotherapies will form part of the treatment programme [107].

*How is the EML4 part of the fusion significant?* In our view, the presence of a misfolded, partial TAPE domain in most EML4-ALK variants is a defining feature of these oncoproteins. This feature underlies exquisite sensitivity to Hsp90 inhibitors and the exposure of the HELP motif promotes RAS signalling. While EML4-ALK v3 localises to microtubules, the presence of a partial TAPE domain in other variants prevents microtubule association and may confer localisation to discrete cytoplasmic structures. This may contribute to oncogenic signalling by promoting co-localisation with other signalling molecules. Further work is required to elaborate the contribution of the EML4 portion of the fusion protein and exploit this in the clinic.

*Will the treatment of EML4*-*ALK patients be stratified by variant type?* EML4-ALK variants are inhibited differently by ALK and Hsp90 inhibitors in vitro, but it remains to be seen whether these differences will be observed in the clinic. However, given the gross differences in the molecular properties of some variants, such as v3 or v5 compared with the others, it seems likely that some differences will be observed in patient response and drug resistance mechanisms. Most patients harbour one of the three most common variants and, provided that the variant is determined during clinical trials, it will be possible to identify any differences of clinical significance. However, it will be difficult to obtain sufficient data for the rare variants and we believe that it is crucial to develop models that provide a rational basis for planning the treatment of these patients.
